# Using Learning Theories to Develop a Veterinary Student Preparedness Toolkit for Workplace Clinical Training

**DOI:** 10.3389/fvets.2022.833034

**Published:** 2022-04-07

**Authors:** Jennifer Routh, Sharmini Julita Paramasivam, Peter Cockcroft, Vishna Devi Nadarajah, Kamalan Jeevaratnam

**Affiliations:** ^1^School of Veterinary Medicine, University of Surrey, Guildford, United Kingdom; ^2^Division of Human Biology, School of Medicine and IMU Centre for Education, International Medical University, Kuala Lumpur, Malaysia

**Keywords:** learning theory, preparedness, preceptorship, veterinary education, workplace learning, clinical rotations

## Abstract

Learning theories are abstract descriptions which help us make sense of educational practice. Multiple theories can inform our understanding of a single concept, in this case: veterinary workplace clinical training (WCT), which occurs just prior to students' graduation as competent veterinary surgeons. The competency movement has strongly influenced reforms in veterinary education and is considered important. In reflection of this, the term “preparedness” is operationalised here as a measure of the likelihood that the veterinary student is going to be a competent learner and participant during WCT. Preparedness itself is therefore important because it directly impacts performance. Workplace clinical training is explored through the lenses of cognitivist, social constructivist and socio-culturalist learning theories and used to inform student preparedness characteristics (“tools”) in terms of their behaviours, personal attributes, knowledge and skills, and awarenesses to optimise learning and participation. These form a new conceptual framework—the “Preparedness Toolkit.”

## Introduction

Veterinary workplace clinical training (WCT) comprises an essential part of the veterinary curriculum and typically takes place in the final 12–24 months of the programme. A form of preceptorship (1), students learn through authentic but supervised participation in “real-world” veterinary practice. Workplace clinical training consists of experiential, informal and opportunistic teaching approaches, albeit guided by structured objective setting and assessment frameworks. It can be located in a number of different types of facilities including university-owned teaching hospitals and clinics, or private practice as part of a distributed curriculum. Often several different types of facility are used for WCT within a programme. Through the integration and application of knowledge, alongside the development of key skills, attributes and professional identity, students transition to competent veterinarians. The development of competency during WCT is critical given that veterinary graduates are licensed to practice unsupervised when they leave the programme, albeit with structured support. Veterinary institutions must therefore ensure that graduates gain the competences required to meet the standards of their statutory registration body to ensure they are able to practice safely and professionally.

Workplace-based medical education has been identified as a “black box” (2); the output is clear but the inner workings are difficult to understand. In a similar way and having developed haphazardly from apprenticeship principles, our direct understanding of how veterinary students learn during WCT is imperfect. In addition, there is limited use and application of learning theories in clinical veterinary education (3–8) which is not representative of their potential usefulness in understanding education practices (9). Indeed, Schoenfeld-Tacher and Baker (10) assess that it's no longer appropriate to expect a clinician to be an outstanding teacher without the appreciation of the underlying principles of learning. As such, theory and practice are linked in a bidirectional manner; theory has the potential to inform practice and to be informed by it (11).

Using learning theory to open the lid of the “black box” and understand the underlying pedagogy of WCT could help us to prepare students for their experiences. Improving preparedness, defined here as “are you going to be a competent learner and participant of WCT?” (12), should improve student competency and produce veterinarians better able to serve the needs of society.

## Learning Theories

A theory is a set of interrelated propositions, concepts or definitions, which specifies the relationships between them, with the aim of explanation or making predictions (13). Learning theories are abstract descriptions which help us make sense of educational practice. More broadly, “conceptual frameworks represent ways of thinking about a problem… or ways of representing how complex things work the way they do” [(14), p. 313]. Different conceptual frameworks can be used to illuminate or magnify different aspects of a system, such as an education programme (14). However, no one conceptual framework can adequately explain or justify an entire system. It is therefore both “necessary and confusing” [(15), p. 70] to acknowledge that there will be many appropriate conceptual frameworks applicable to any topic of interest. The conceptual frameworks cognitivism, social constructivism and socio-culturalism encompass different learning theories and focus on different ways of understanding learning in the workplace. The ways in which a range of these learning theories align with WCT will be discussed in this paper and the aim is to produce a new conceptual framework to model how students might be prepared for veterinary WCT.

### Cognitivist Theories

Cognitivism focuses on individual psychology and in the context of workplace learning is concerned with the epistemology of professional practice, in other words how do we know how to be a veterinary surgeon? As such, its explanatory power is limited to the individual student. Cognitivism endeavours to explain how knowledge and skills are acquired through transmission (with the input of another resource) and constructivism. Constructivism is important to consider in the context of WCT. It describes how knowledge, meaning or learning is “constructed” and integrated into an expanding but existing network with experience. Nothing is learned from scratch; knowledge is assimilated or reinterpreted in the light of the learner's existing understanding and ability (16). The influence of constructivist concepts such as andragogy (17) and reflective learning (18) is exemplified by the adoption of portfolios, personal development logs and learning objective planning in veterinary curricula.

#### Kolb's Learning Cycle

Kolb's learning cycle combines the models of learning developed by Dewey, Lewin and Piaget (16). The cycle describes how knowledge is continuously derived from and tested out in the experiences of the learner, and Kolb is credited with proposing that reflection is how learners abstract and transfer their learning to new contexts. In the constructivist style, Kolb states that all learning is re-learning; learners' minds are not blank sheets of paper. This aligns readily with veterinary students entering WCT, their sheet of paper already populated with some concepts (for example their knowledge of anatomy, pathology, microbiology). It is by their experiences in the workplace that students learn how to integrate and apply these subjects in the practice of veterinary medicine.

The learning that Kolb describes is more extensive than that which occurs in the confines of the lecture theatre; learning occurs in all settings (including the workplace) and is lifelong. Additionally, it encompasses not only knowledge acquisition, but the cycle can be applied to other cognitive concepts such as decision making and problem-solving. Learning is viewed by Kolb as a holistic process and involves thinking, feeling, perceiving, and behaving. All these facets of the theory make it attractive for application to veterinary WCT (Table 1).

**Table 1:** The stages of Kolb's learning cycle, applied to veterinary workplace clinical training (WCT) and inferred student preparedness characteristics.

**Stage of Kolb's learning cycle**	**Applied to veterinary student workplace clinical training (WCT)**	**Inferred student preparedness characteristics**
Concrete experience	Students must gain experience in many clinical areas as part of their WCT. The more granular experiences expected may be prescribed in a list for students prior to commencing, e.g., the student is assigned to a real patient, takes the clinical history and performs the clinical exam.	Students should be prepared to immerse themselves fully in the activity of the workplace to have concrete (i.e., authentic) experiences. Individuals need to harness the opportunities that arise in the workplace, which may be on an *ad hoc* basis. As such students will require an awareness of what their potential experiences are and what they can get out of them—without preparation students may overlook opportunities to learn.
Reflective observation (reflecting on the experience)	The student “makes sense” of what they experienced; they reflect on the encounter. This may be triggered by a clinical supervisor in feedback dialogue or in the writing of a reflective report.	Students should be proficient at reflection and recognising their experiences from other perspectives. They should be proactive and seek formative feedback in order to facilitate their reflection (19); it should not be assumed that feedback is part of every practice culture.
Abstract conceptualisation (generating new approaches)	The student uses the reflective process to generate new approaches to self-improvement; what they need to learn to build on existing knowledge or improve techniques. This could be evidenced in a written portfolio or through the setting of personalised learning objectives.	To be well-prepared for WCT students should be capable in self-directed learning.
Active experiment (testing new approaches)	The student tests their new approach in their next experience for example, new interviewing skills, clinic exam techniques or approaches to clinical reasoning. The result is that students gain the skills or knowledge required within the clinical area.	Students need to plan new actions to promote further experiential learning, this could include discussions with workplace supervisors to arrange upcoming opportunities.

Greenberg and Blatt (20) provide a guide for medical students in negotiating their undergraduate WCT equivalent, based on Kolb's learning cycle. They propose a preparatory fifth stage suggesting that students should learn and be prepared to implement adult learning principles and Kolb's experiential learning cycle. They state that students should identify their roles and responsibilities prior to commencing training to give them confidence in seeking out concrete experiences. Students also need to understand that they must act within their boundaries and to be certain that clients understand their level of training; experiences must be appropriate for the training level.

#### Schön's Reflective Learning Theory

Schön's reflective learning theory (18) specifically addresses learning in contexts of uncertainty, working in the “swamp” with messy and confusing problems. The veterinary workplace is swamp-like, it can be unpredictable and difficult to see a clear path to take to solve patients' problems. Clinical cases presented are often not as the textbook would describe but are unique and might require improvisation. Schön theorises that practitioners work in the “indeterminate zones of practice,” characterised by this uncertainty, uniqueness, where values might conflict, and “technical rationality” can fall down. Schön developed the concept of the “reflective practitioner” to help students acquire the kind of artistry essential to professional competence in these indeterminate zones of practice.

Firstly, we consider “reflecting in action,” where, without interrupting action, thinking reshapes the action. “Reflecting in action” can be likened to trial and error, but successive trials have an immediate significance for the next action; they are iterative, and trials are not performed at random. A good example would be a veterinary student making attempts at venepuncture, redirecting the needle each time based upon their quick reflections in the action. Students will also need to be prepared for performing the on-the-spot experiments of “reflecting in action” in the workplace by putting their knowledge to the test in other contexts. An example would be the recognition of clinical signs or proposing treatment options for a disease known in one species to another.

Secondly, we consider “reflecting on action,” the “stop and think” phase, where practitioners reflect later, after an event. Schön explains that it is crucial that a learner thinks about the processes that they have used and the extent to which they were appropriate. In the veterinary workplace reflecting on action can be facilitated using reflective journals or portfolios in addition to feedback from supervisors.

Schön theorises that reflective practice (both in and on action) becomes a vehicle for learning effectively in the professional environment and may be integrated into future “knowing in action” or “professional artistry” (11). Students will need to graduate omnicompetent not omniscient, it is not possible to know everything (21), and reflection is key to managing this insurmountable challenge. In other words, without reflection contextual learning will be trapped in the precise context in which it was learnt. Reflection is evidently something that students must be prepared for.

#### Knowles' Andragogy

With roots in humanistic psychology, the foundation of Knowles' somewhat controversial andragogy was initially to distinguish between the ways in which children and adults learn (pedagogy vs. andragogy) (17). Many authors have argued that the dichotomisation of andragogy and pedagogy is unfounded, lacking a conceptual basis and empirical evidence; children and adults do not necessarily learn differently because of their age. Andragogy is presented as a series of assumptions about the adult learner (Table 2), and it is argued by some that it's neither a true learning theory nor a proven educational method (4, 22, 23). Nonetheless, andragogy is stated in terms of the learners' behaviour as opposed to teachers' actions, and therefore could be utilised to infer how students might be prepared for WCT.

**Table 2:** The assumptions of andragogy applied to veterinary workplace clinical training (WCT).

**Andragogy describes the adult learner as…**	**Applied to veterinary WCT**
Independent and self-directed	Andragogy is fostered by a curriculum strongly focused on self-directed learning which, in the absence of formal didactic teaching, instinctively aligns well with veterinary WCT.
Having accumulated a reservoir of life experiences that are a rich source for learning	Both through their life pre-training and their university career (for example through participation in extra-mural studies in the UK), students will have gained their own knowledge, experiences and expectations, which they bring to the workplace.
Having learning needs closely related to changing social roles	In the clinic or hospital students can clearly relate their patient-centered work and learning to their changing
Problem centered and interested in immediate application of knowledge	societal role as a veterinary professional.
Motivated to learn by internal rather than external factors	In the final year as graduation draws closer, students are internally motivated by the need to become competent practicing veterinarians, which can be promoted by self-selected placements (electives). This is provided that curriculum overload or overwhelming assessments do not force students to revert back to rote memorisation of facts in order to keep up or pass.

In work by Matthew et al. (24, 25) a *cohesive* conception of WCT learning by veterinary students (learning the contextual variation that affects the management of cases in real-world veterinary practice) was associated with *deep* learning approaches. These approaches were strongly andragogical; they emphasised independent, reflective and self-directed learning alongside intrinsic motivation and satisfaction in progressing towards competency in practice. Veterinary students adopting such an approach performed better in assessments of WCT, which were an indicator of practice readiness. This provides evidence of the importance of these andragogical approaches, which students should be prepared with.

In an “adult” classroom, learners should feel accepted, respected and supported with a spirit of joint enquiry between teachers and students (17). Therefore, in addition to internal motivation and self-directed learning skills, students should be prepared for the dynamics of the relationship with their educators to change with the transition to WCT. Instead of being presented with information by lecturers, students should feel prepared to be part of the clinical team, solving problems alongside their teacher clinicians.

#### Threshold Concepts

When considering student preparedness for WCT, Meyer and Land's threshold concepts offer promise as a useful tool (26, 27). Likened to a portal, threshold concepts are ideas that are necessary for a student to possess in order to progress in their learning, and eventually enable them to think like a professional. They are transformational and change the way that the student thinks about a concept, not just what they know about it. Threshold concepts could be thought of as keys to preparedness for the next stage of learning, or which allow students to participate effectively in the workplace.

Attempts have been made to examine threshold concepts in veterinary education (28). Pharmacology and neurology areas were identified as the most challenging subjects across the entire curriculum in two separate schools, which is part-way towards classification as a threshold concept. However, the study was unable to determine any areas that once understood transformed students' way of thinking or professional development, and therefore could not identify any legitimate threshold concepts.

**Figure 1:**
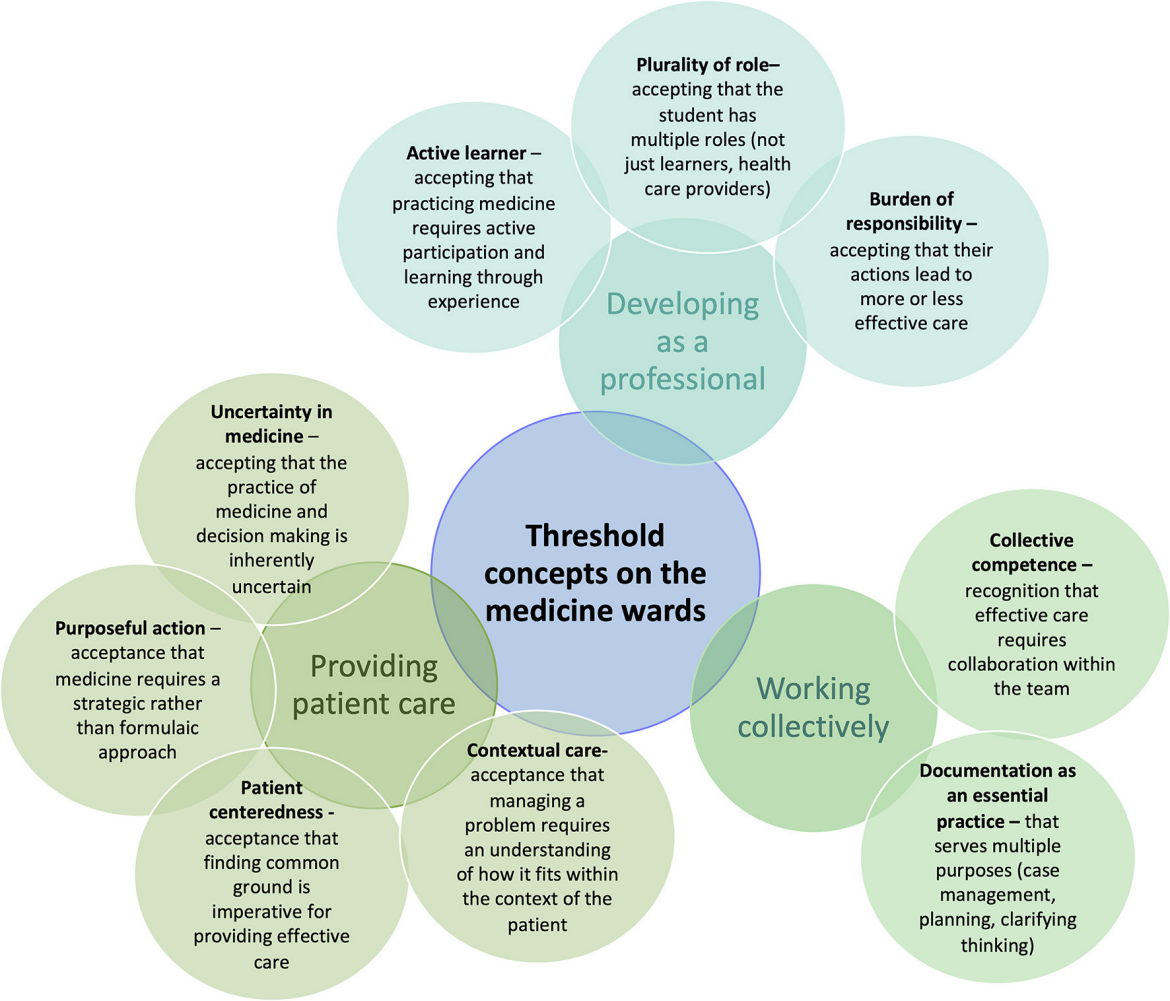
Threshold concepts encountered by junior trainees on an internal medicine rotation, content from Bhat et al. (29).

Threshold concepts have been assessed in human medicine (29–31). Bhat et al. (29) describe nine threshold concepts that junior trainees may encounter during an internal medicine rotation (Figure 1). Additionally, working with uncertainty, considering the bigger picture, not needing to know everything and professional culture have been identified as threshold concepts for professionalism learning in undergraduate medicine (31). These could be considered as candidate preparedness characteristics for veterinary WCT.

Gaunt and Loffman (32) explain that medical students should be prepared to seek out opportunities to tackle threshold concepts. If students understand that by accomplishing threshold concepts, they will unlock new ways of thinking previously closed off to them, it will create more confident and curious learners with a professional mindset that can succeed in the workplace.

## Social Constructivist Theories

Like cognitivism, in social constructivism focus remains on the individual's internal mind. However, social constructivism contrasts with the independent learning embedded in much of the cognitivist theories and formal, classroom based, veterinary education; these theories explore how learning can be shaped by the engagement of experience with others.

### Vygotsky's Zone of Proximal Development

Through his work to describe the zone of proximal development (ZPD), Vygotsky is a key contributor to social constructivism (33). He depicts the ZPD as the gap between (1) a students' actual developmental level, which is their proven, independent mental function and (2) the level of potential development which can be established by problem-solving under the guidance of, or in collaboration with, more capable others (33).

There are three key features of the ZPD theory that can be taken forward into WCT (34). Firstly, the use of “tools” to “mediate” learning. Using patients as a scaffold for learning is the central tenet of WCT and they are fundamentally a teaching tool. Using diagnostic images, laboratory results or case notes would be other physical examples, but tools can also be symbolic such as mnemonics to help remember differential diagnoses. Secondly, identifying learners' actual developmental level and their learning potential. Students could prepare for their placements by considering and even communicating their actual developmental level, and identify gaps in their knowledge or skills, which is expected by veterinary clinical supervisors (35). Thirdly, students should prepare to learn from the entire clinical team. If one locates learning in the social arena of a workplace, a whole community (veterinary surgeons, nurses, receptionists) opens up to assisting with navigation across the ZPD. The value of near-peer education also aligns well with the social nature of Vygotsky's theory, particularly when considering preparing for transitions to new working environments. The “student grapevine” (acquiring information from students who have previously experienced that WCT environment) has been identified in both the medical (36, 37) and veterinary (35, 38) settings.

## Socio-Culturalist Theories

Learning in complex and dynamic systems such as the veterinary workplace cannot be wholly explained by cognitive or constructivist theories alone and is perhaps best understood from a socio-cultural viewpoint. Socio-cultural learning theories underlie the learning-as-participation metaphor (as opposed to learning-as-acquisition) (39). The goal is the full, authentic participation in the work of a community, which has a striking resemblance to the aims of veterinary WCT.

### Situated Learning Theory

Lave and Wenger (40) conceived situated learning theory which draws together the learner, their activity and their world as “mutually constitutive.” Learning is a process of participation in “communities of practice,” that is “legitimate” and at first “peripheral” but that gradually increases in engagement and complexity. The learning is embodied in the everyday activity of the community and knowledge is co-constructed through participation and social interaction. An expansion of these key terms in situated learning theory are presented in Supplementary Item One (40, 41).

**Figure 2:**
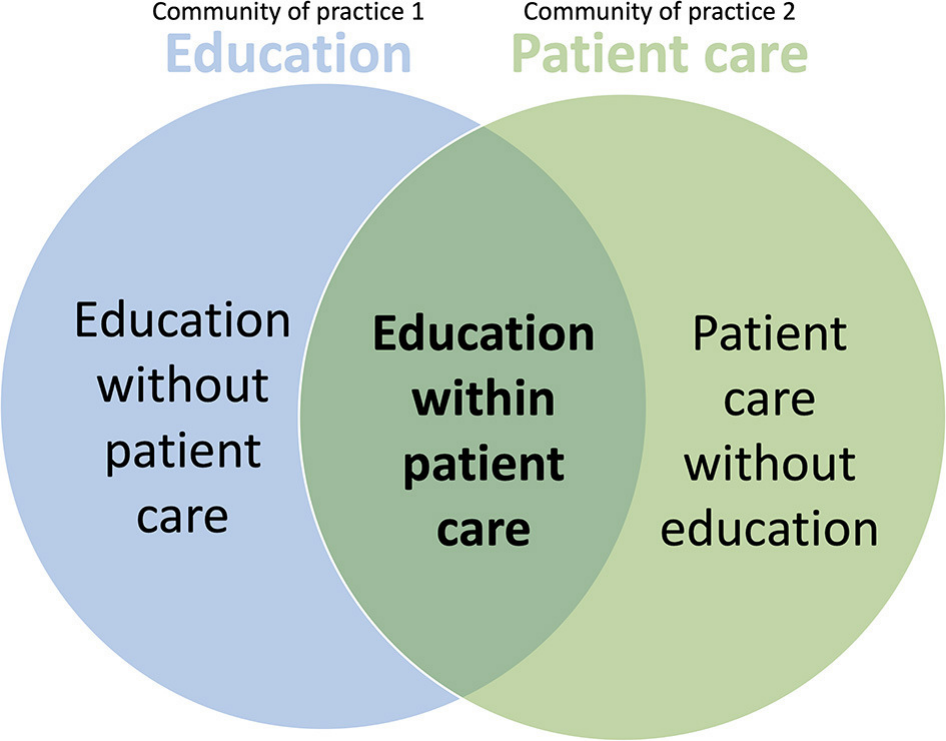
The two communities of practice that medical clerkship students participate in (education and patient care). Situated learning occurs when patient care created learning opportunities, which were enriched when practitioners intentionally supported participants' learning (42).

Steven et al. (42) demonstrated that medical clerkship students learn from real patients by participating in patient care within an educational practice. The students participated in two communities of practice: education and patient care (Figure 2). In some cases, clinical care was provided but no teaching took place and students were unable to learn from the practice, in other cases, students learnt and/or were taught but patient care was not contributory. In education within patient care, students learnt from patient care without intentional teaching. This represents situated learning. In a similar way, undergraduate veterinary students are assigned to various teaching sites (and communities of practice) throughout their clinical training where they are immersed in the work of patient care, to a greater or lesser extent.

How clinical students learn from real patients in veterinary workplace settings has not been examined prospectively through the lens of situated learning theory but is discussed from a veterinary perspective by Scholz et al. (5). Despite the lack of research, there are facets of veterinary WCT which seem to align well with situated learning theory. First and foremost, veterinary students should broadly be legitimate peripheral participants in a community of practice during WCT (5). Secondly, it is recognised that veterinary students learn from the formal (intended and scheduled), informal (opportunistic) and hidden curricula of the veterinary workplace (43–47). The hidden curriculum is the “underbelly” of practice and training (47), the unwritten information that is tacitly absorbed by students from their environment, particularly what it's like to be a professional in the workplace (enculturation), which is a central tenet of situated learning. Thirdly, students do have increasing levels of responsibility in the workplace as learning occurs and trust develops within the community of practice and they should be genuinely regarded as part of the team. They demonstrate what they have learned through exhibiting competency, externalising rather than internalising knowledge. In an ideal world, veterinary schools would like students' participation to be authentic, and they should take part in all kinds of activity. They should not be “sequestered” away and left to learn abstract knowledge.

Steven et al. (42) describe how practitioners supported students' learning and verbal communication was identified as critical for this. The nature and quality of learning were dependent on clinicians entering a dialogue with students linked to patient care. Scholz et al. (5) and King et al. (48) also highlight the importance of language in veterinary WCT, which is fundamentally a social enterprise. Participants learn both the technical terminology of the sciences alongside the tacit tone and language required for communicating successfully with clients and colleagues in various contexts, for example on the farmyard, in the consultation room, in theatre or the staff room. The importance of language and the way in which WCT students use it is reflected in the emphasis now placed on communication skills in modern veterinary curricula (49–53).

Situated learning theory provides a detailed model for how students might learn during WCT and there are several facets from which suitable student preparation can be deduced, which are discussed in Table 3.

**Table 3:** How can we use situated learning theory to inform valuable student preparedness characteristics for workplace clinical training (WCT)?

**Situated learning theory**	**Inferred student preparedness characteristics**
The student is “decentralised” in the community of practice, where the focus is on the work to be performed.	Students should understand patient vs. student centered education. They should expect priority to be placed on the work of the practice and not necessarily on their teaching. Students need to be prepared to seek the opportunities to learn in all situations; it is likely that they will not be made explicit.
Situated learning is taking part in all kinds of activity of the community of practice.	Students should be prepared to “muck in” with all types of work whilst participating in WCT and this might include tasks traditionally not ascribed to veterinarians such as patient restraint or cleaning. Even if not obviously applicable to their personal learning objectives students should understand that all kinds of activity enable them to learn about the culture of the community of practice. Key student attributes for this could include humility and being a team player.
Students require a “developmental space” to be able to learn and develop their professional identity (54).	The development space constitutes (54): 1) the attributes of the practice (such as computer system access)—orientation or transition courses might help students familiarise themselves with these aspects (55) 2) productive relationships with other community members—interpersonal skills are likely to be important 3) students' state of mind; feeling motivated and self-confident are emotional pre-requisites for the creation of developmental space and the subsequent opportunities for personal growth
By legitimately contributing to the community of practice, students can develop a view of what the whole enterprise is about, which can act as a strong motivator.	Knowing about the community of practice will only be motivating provided that the student is intrinsically motivated (56), i.e., they are learning in order to become a good veterinarian, as opposed to being extrinsically motivated to simply pass examinations.
Rather than a student/teacher dyad there is a richly diverse field of people to learn from in the community of practice.	Students need to be prepared for interprofessional and peer to peer education. This may be particularly useful when there is “benign neglect” by more experienced and central members of the community of practice. Students need to appreciate that they can learn from all members of the professional team. Additionally, “teachers” fill multiple roles and at times working and teaching may be in conflict.
Students are peripheral participants, at first.	Students should have realistic expectations of the peripherality of their participation as new clinical trainees, and the expected centripetal “journey” to the core of the practice where they are competent veterinarians by the end. The value of the learner to the community of practice increases as they become more proficient, which should provide intrinsic reward.
Students are treated as newcomers and not necessarily novices in the community of practice.	It is expected that students will enter the community of practice with some pre-existing knowledge and experience. Students could do well to consolidate this prior to commencing clinical training. As newcomers, students can contribute to the community of practice in meaningful ways. Such responsibility and level of professionalism expected from students aligns with the Royal College of Veterinary Surgeons' description of “nearly professionals” in the workplace (57).
Context is an integral part of practice and its activities (5), which in turn affects the affordances (opportunities) for learning.	Students should approach workplace learning with an appreciation of the context in which the community of practice is situated. For example: The seasonal context: the type of work performed at a farm practice will be dictated somewhat by the time of year, which determines learning opportunities for students. The social context: for example, students' knowing which staff member organises the students into farm vets' cars for visits. The economic context: the socio-economic background of the practice and/or clients' insurance status for their animals will impact the complexity of diagnostics and treatment students will encounter.

Situated learning in communities of practice has developed greatly since its conception (58). This occurs frequently with learning theories, which aren't static, but are tested, refined and evolve. As an example, Wenger-Trayner et al. (59) introduced the concept of a “landscape of practice” as a system of communities of practice. An example would be the veterinary profession with its constituent communities of practice (veterinary practices, regulatory bodies, veterinary schools, research communities). Professional “knowledgeability” has been introduced as the ability of a practitioner to transform what they know about all the different communities of practice to produce meaningful action (for example the delivery of veterinary care) in their own community of practice. If knowledgeability is valued in our landscape of practice, this could have implications for inter-professional education (60), professional identity formation and evidence-based veterinary medicine. Although this development in situated learning theory doesn't intuitively inform preparedness for WCT, it does highlight the requirement for educators to be alert to these developments and their implications for their practice.

### Cultural Historical Activity Theory

Cultural Historical Activity Theory, which is also referred to as activity theory or CHAT, focuses on goal-directed joint activity (22). In this context, activity is not only what and how the subject is performing, but why (termed the object). A key way in which it is different to the other socio-cultural theories is that the intended goal of learning does not align well with Sfard's learning-as-participation, nor learning-as-acquisition metaphors (39, 61). Instead, the goal is the expansive transformation of systems; deliberate change that results from tensions arising within a system.

Activity theory takes root in Vygotsky's triad of human-mediated activity in which “activity” represents the basic unit of analysis (33). The triad consists of a subject (the student, the veterinary surgeon), mediating tools (e.g., patients, diagnostic images) and an object (e.g., preparing future veterinarians, treating patients). In its second-generation form, Engeström describes activity theory as a conceptual framework for describing the interactions of people, the tools that they use and the rules within complex “activity systems” (62–64). The key terms of activity theory are expanded in Supplementary Item Two (62–64).

The third generation of activity theory exists where multiple activity systems interact and compete (62, 65, 66) (**Figure 5**). An example would be the activity systems of the classroom-based veterinary school and the clinical teaching facilities that host students. Whilst participants have a shared outcome (veterinary education), the ways in which they mediate their activity and the communities in which they occur are different. Preparedness for the transition from one activity system to the other (“boundary crossing”) should consider to what extent students can make sense of the teaching practices used in each system, and identify and react to the changing cultures and rules in their new environment (68). We can consider boundary-crossing artefacts, e.g., communication skills practice using actors in the classroom, or clinical skill simulation in the workplace, as potentially useful in preparing for this transition (68).

**Figure 3:**
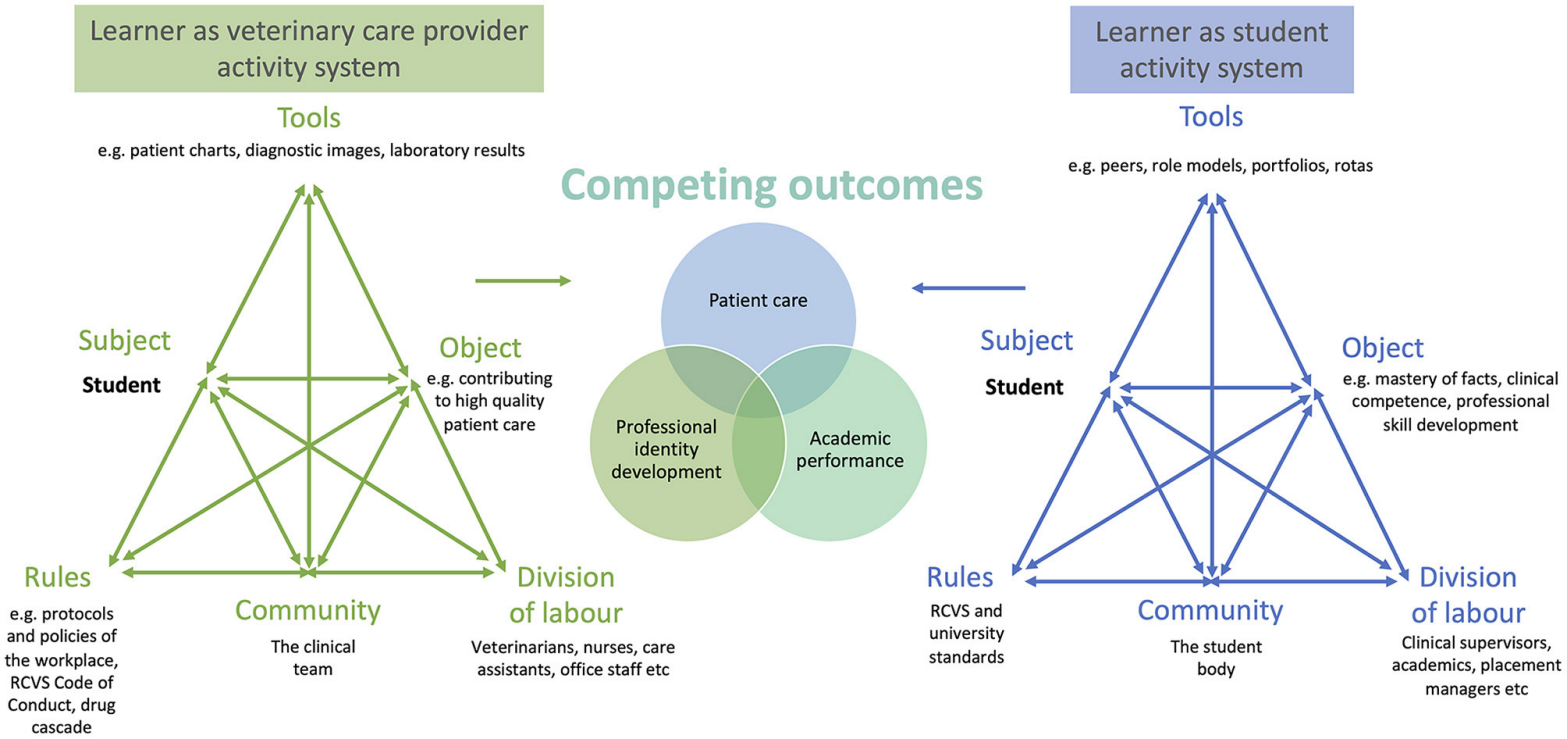
Adapted with permission from Larsen et al. (65) for the veterinary WCT context. The clinical veterinary student in the workplace is part of several activity systems, between which there may be interaction or tensions and competing outcomes. RCVS, Royal College of Veterinary Surgeons.

Another relevant example of two activity systems in competition would be “student as learner” and “student as veterinary care provider” systems. When veterinary students are both working and learning in the workplace these activity systems have overlapping but not fully aligned outcomes (Figure 3). An instance of tension would be when a student has the desire to develop a specific skill (object) in order to become a competent veterinarian (outcome), but they do not want to cause the patient or client undue inconvenience by practising with them (a competing object in a different activity system, with the outcome of patient care) (35, 66). Simultaneously the student may feel pressure to display their competence in the skill to the clinical supervisor to achieve a good assessment result (another outcome).

However, these tensions are creative forces for change (69). To overcome them, new ways of acting or new objects can be developed in the workplace and this process is termed expansive learning (70). In the above example, the clinical supervisor could help the student to identify amenable patients to practice with and facilitate getting consent.

Using written learning goals for clinical workplace training has been described as a tool to overcome tensions in the medical workplace (65, 66) and several desirable preparedness characteristics could be assumed from this work. Larsen et al. identified that student engagement with and commitment to goals were important and that students should understand that they can use learning goals to negotiate the demands faced in the workplace. This may include prioritising who or what they observe and take part in, reflecting on how their time is best used and acknowledgement that a degree of proactivity is required to create opportunities for participation. However, the vicissitude of daily practice needs to be acknowledged and a realisation that the learning goals of the student will not necessarily match the needs of the patients that walk in through the door. Some supervisors identified broader goals to be useful; these were seen as tools for leveraging greater learning opportunities because as they fit many more scenarios.

### Experience Based Learning

**Figure 4:**
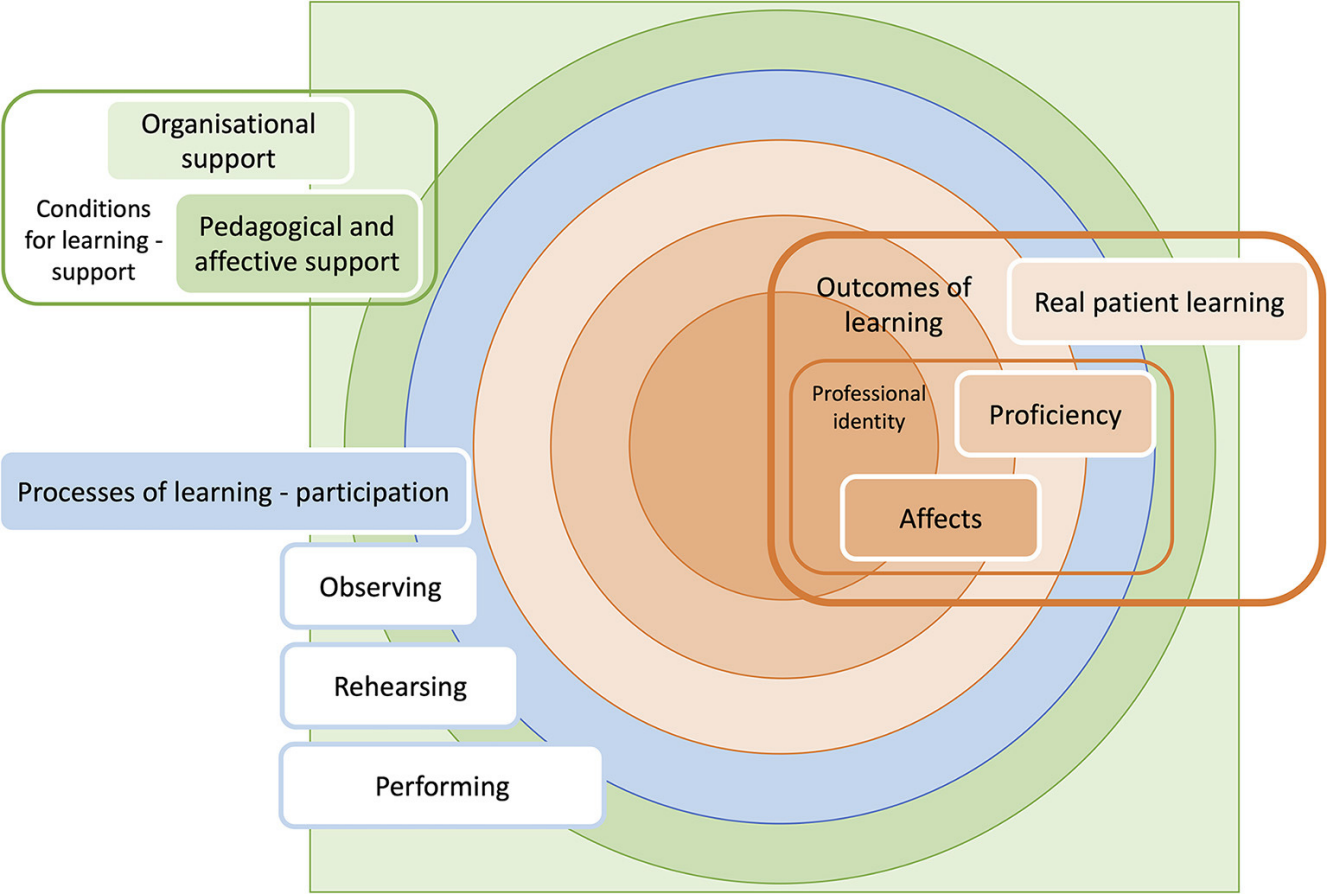
Experience based learning theory adapted with permission from Dornan et al. (71).

Dornan et al. explored the pedagogic principles that underlie rotation based medical clerkships (analogous to veterinary WCT), and from it developed Experience Based Learning (ExBL) theory (Figure 4) (71, 72). The theory clearly draws on the higher communities of practice theory from Lave and Wenger (40) but is applied to the clinical setting resulting in a middle-range theory grounded in empirical findings.

The theory states that medical students learn during clerkships as a result of “supported participation in practice” (71, 73). Participation is embedded within triadic relationships between the student, clinical supervisors, and their patients in authentic workplaces. Within the triad, the student can have an observatory (passive or active) or actor-like (in rehearsal or in performance) role (Figure 4). Unlike in Lave and Wenger's (40) communities of practice, teaching is observable and does contribute to ExBL, however, it is not a central or defining feature. Participation is always supported, and it is provided in three ways: pedagogic, affective, and organisational support (Figure 4). Participation is rooted in “real patient learning,” a critical process that results in both practical (proficiency) and affective outcomes, but it is also a learning outcome in itself. These outcomes contribute to professional identity development. How these outcomes can be used to inform the preparation of veterinary students for “supported participation in practice” is discussed in Table 4.

**Table 4:** How can experience based learning (ExBL) theory outcomes be used to inform valuable preparedness characteristics for veterinary workplace clinical training (WCT)?

	**Learning outcome** **(****42**, **72****)**	**Inferred student preparedness characteristics**
Real patient learning	• A reflective process • Results from the interaction with real patients • Involves linking existing knowledge to memorable patients, i.e., restructuring, consolidating, reinforcing, and contextualising prior learning • Involves integrating knowledge with attitudes and skills • Contributes to a students' ability to care for a patient • Allows students to gain the quantity of experience and hours of supervision to become competent	• Skills in reflection (on oneself, one's reactions to others, professional identity, capabilities, aspirations) • Sound theoretical knowledge in basic sciences, common conditions, and therapy practices • Foundation skills for that area of practice • Strong commitment to the workload and working hours demanded of students
Affective learning	• Includes students' motions, moods, state of mind • Can be directed at oneself or towards others • Positive: a sense of belonging, increasing comfort, legitimacy, coping with uncertainty, sense of satisfaction and reward, empathy and compassion • Negative: illegitimacy, discomfort assuming the role of a student doctor, loss of confidence, demotivation, frustration, anxiety • Negative emotions were often associated with transitions when students struggled to adjust—this has potentially important implications for preparedness	• Ability to recognise and respond appropriately to negative moods states • Ability to recognise and respond appropriately to negative emotions directed at the student (being non-judgmental, respectful, empathetic) • Recognising boundaries and limits • Strong motivation, demonstrating a desire to learn, enthusiasm and interest in the training
Practical learning (proficiency)	• Learning how to practice—understanding it, rather than simply knowing about it • Includes more traditionally recognised learning outcomes—knowledge and practical skills • Organisational aspects of practice • How to manage learning—identifying individual limits and then establishing learning needs, and where to find then use resources	• Knowledge of other professions and their roles in the shared workspace • Some understanding of the department/ organisation where they will be undertaking training • Ability to adhere to workplace standard operating procedures and policies • Knowledge of how to access information when a gap in knowledge is identified • Understanding of their own learning style

### Organisational Socialisation Theory

Organisational socialisation theory describes the way in which newcomers move from being organisational outsiders to organisational insiders (67). It incorporates the process by which new members learn the knowledge, skills and behaviours they need to succeed in their new environment. It is distinct from the process of *occupational* socialisation which describes professional identity formation. Organisational socialisation theory is important to consider because students are embedded in social organisations during WCT and the transition into it reflects the effectiveness of the programme in preparing students.

**Figure 5:**
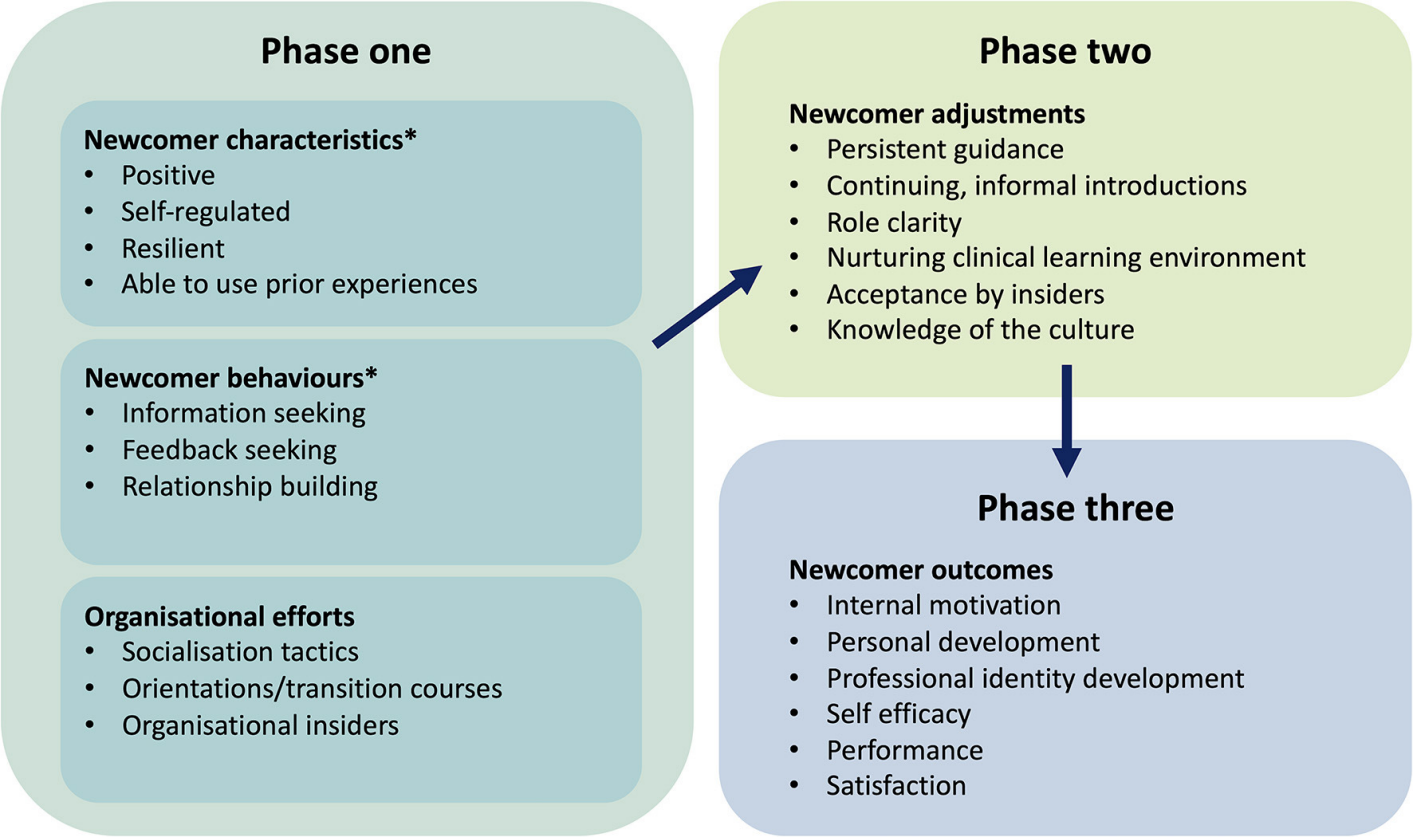
Organisational socialisation theory. Adapted from Atherley et al. (37) and Bauer & Erdogan (67). *Newcomer characteristics and behaviours align particularly well to preparedness for workplace clinical training (WCT).

Organisational socialisation theory has been utilised by Atherley et al. (37) to explore how medical students transition within their rotational clinical training, additionally it has been applied to nursing graduates entering the clinical workplace (74). The first phase of the theory has three components (67): newcomer characteristics, newcomer behaviours and organisational efforts. This is followed by an adjustment phase, leading to newcomer outcomes (Figure 5).

Newcomer characteristics and behaviours directly align with preparedness for entering the workplace. Through student focus groups and open-ended written questions, Atherley et al. (37) found that students recognised the need to commence a placement with proactivity, positivity and the ability to build productive relationships. Students were required to ignore the reputation that preceded the rotation [*via* the “student grapevine” (36)] to achieve this, or risk anxiety prohibiting learning. Peer-to-peer preparation for veterinary WCT has also been identified as conceivably problematic given the potential for the transmission of misconceptions (35). Many students were concerned about their role as part of the medical team and clarification would have been useful in preparation. Only a few students looked forward to feedback-seeking but those that did thought it would be useful in the transition. It was also suggested that targeting skills such as self-regulated learning [goal-setting, self-monitoring and self-reflection (75)] and resilience in the pre-clinical phase may prove useful in developing proactivity and feedback-seeking behaviour.

### Billet's Theory of Workplace Affordances and Individual Engagements

Billet's theory of workplace affordances and individual engagements identifies the factors that shape learning in workplaces (76–78). It is centered on co-participation, and the duality of how the workplace invites access to activities and guidance (workplace affordances) but also how individuals elect to participate in what the workplace affords (individual engagements).

King et al. (48) developed a constructivist grounded theory to understand how veterinary students interact in the workplace and Billet's workplace affordances and individual engagements provided a conceptual orientation for the work. The resulting theory “Learning to Interact and Interacting to Learn” (LIIL) has a central concept of harnessing dialogue. Students came to understand that the way in which they engaged in dialogue influenced three inter-related processes (1) contributing to the daily functioning of the workplace, (2) the impressions that others had of them and (3) learning in the moment, acquiring specific skills or knowledge whilst working. They established that some students had to learn to interact more effectively, to be able to learn from interactions. Preparation of veterinary students for learning in the workplace could therefore include building social discourse awareness to enable students to perceive how best to find learning opportunities through interaction.

The work by King et al. shines a light on the importance of learner agency; how veterinary students elect to engage with workplace activities. Billet explains that learning new knowledge is an active, demanding process; individuals must be prepared to engage actively and deliberately seek to improve performance. This process includes self-directed learning, interacting with others, objects, and artefacts, and perceptively seeking clues from the social and physical environment for arising opportunities, and then monitoring progress during task completion.

Additionally, veterinary students might elect to engage actively in some components of activities but less actively (and even resentfully) in others, often simultaneously. It is also likely that individuals' interests and priorities will mediate their participation in work. This is perhaps enhanced by the fact that veterinary students, at the time of WCT, are likely to have species-specific career aspirations, which according to Billet's theory may influence the effort they expend on each rotation. Given that full-bodied engagement by individuals is likely to be required to develop robust vocational practices, we can infer that veterinary students should engage with all opportunities with equal enthusiasm and endeavour, despite any influence of their individual interests.

## Discussion: Inferences About Student Preparedness

No single theory of learning can adequately explain the process of WCT in its entirety or in all its detail, each one magnifies certain aspects of the world, and possibly distorts or hides other aspects. However, by considering a number of theories, principles and models a framework of learning can be constructed, which can be used to inform different components of WCT preparedness and as a reference for discussion. It is important to acknowledge that although the authors have attempted to be wide-ranging in their selection of learning theories to review, it is not complete, and there are other relevant learning theories that one might consider with respect to clinical education (such as Zimmerman's Self-regulated Learning Theory). We should also recognise that these theories describe what should or could happen in ideal circumstances, and it cannot be assumed that every clinical training experience will be like this. Extensive research using objectivist deductivist approaches will be required to confirm how well, or how poorly, the learning theories approximate veterinary WCT in reality (13).

All learning theories have been subjected to critique by the research community, but cognitivism and social constructivism, in particular, have some limitations in providing an adequate account of workplace learning that are worthy of acknowledgement. If only examined through the lens of cognitivism, learning in the workplace would be understood as concerned with only the individual, as an essentially unmediated activity (learners engaging with their own experience with little input from others), independent of context, unproblematic, and with learning treated as a “thing” (which lends itself to the knowledge acquisition and transfer metaphors). Additionally, from the perspective of cognitivism, the performance of work consists of thinking and then the application of this thinking and the role of social or cultural factors are, at best, a background feature of workplace learning, and are underestimated. Similarly, social constructivism still ignores the context in which learning takes place, which we believe to be important. The ZPD ascribes a label to something clinical educators are probably already aware of: that students know what they know and, with their help, can progress to a more advanced level. However, the theory doesn't add detail about *how* this might be achieved. Additionally, like cognitivism, the ZPD remains individualistic and describes teaching on a one-to-one basis and so fails to account for group learning which occurs in the workplace.

It is intuitive that WCT is best understood from a socio-cultural perspective. However, there is very little published putting these theories to work in veterinary education and much of the content discussed here draws on observations and analysis of medical clerkships. It is reasonable to deduce that similarly veterinary WCT is shaped by experience as a peripheral participant within a community of practice. Students can be seen as boundary crossers who are engaged with the objects of two activity systems (student-as-learner and student-as-worker). Through observing, rehearsing and performing students achieve affective and practical learning outcomes alongside real patient learning. Organisational socialisation theory suggests that newcomer characteristics, behaviours and adjustments could help students to transition to workplace learning proficiently. Some aspects of cognitivism are also instinctively important for veterinary clinical trainees: grasping threshold concepts such as patient-centeredness and uncertainty and risk in decision making, reflecting in and on action and conducting their training in an andragogical manner.

Being prepared for WCT and the student's ability to learn is not limited by their actual development level (33); students can possess sufficient prior knowledge but fail to learn in workplaces (79). This is because competence in WCT is likely to be highly contextualised (80); it is fundamentally affected by the activity, organisational practices and culture of that particular workplace (81). In other words, some responsibility for students' competence must be placed on the workplace itself and other members of the community of practice. Preparedness is also individualised and fluctuates over time (82). For these reasons, it is not possible to be *fully* prepared for a transition into WCT, and it should be viewed as a “critically intensive learning period” instead (81). However, by understanding how students learn from WCT using learning theory, we can deduce how students could be placed in the best position to learn from those experiences, i.e., what tools can they possess to be best prepared?

**Figure 6:**
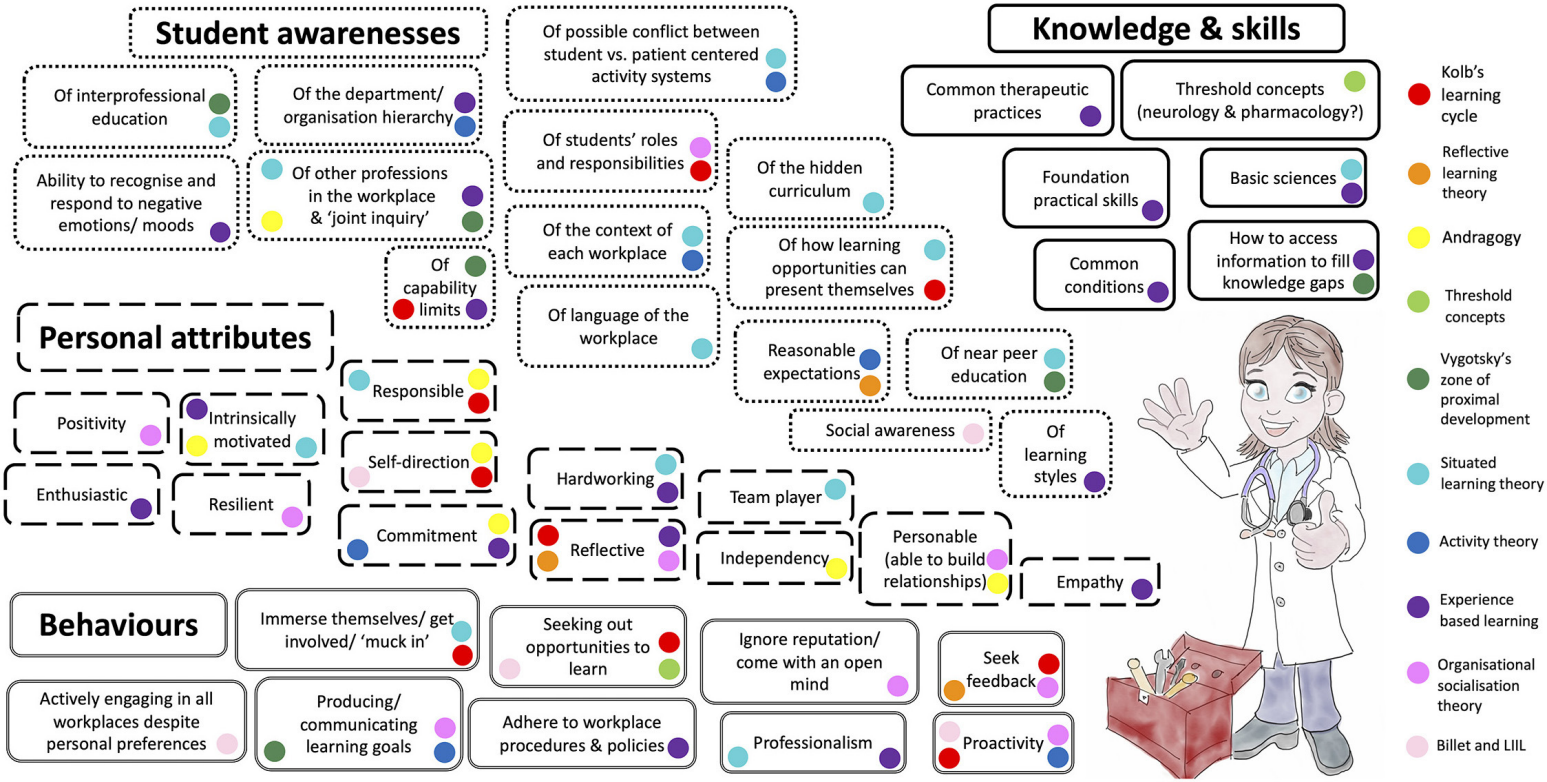
The veterinary clinical trainee preparedness toolbox contains the preparedness characteristics, derived from an understanding of relevant learning theory, which could be utilised to engage with, and learn from, the veterinary workplace by undergraduate veterinary students (LIIL, Learning to Interact, Interacting to Learn).

“A conceptual framework is the end result of bringing together a number of related concepts to explain or predict a given event, or give a broader understanding of the phenomenon of interest” [(83), p. 189]. Using this narrative review we have developed a new conceptual framework, the “Preparedness Toolkit” as a useful way to think about our phenomenon of interest: preparedness for WCT (Figure 6). The “Preparedness Toolkit” contains “tools” derived from the learning theories explored in this paper (as detailed by the colour key). We propose that if possessed and put to work by the veterinary student, these “tools” will assist them in learning and working at the expected level during WCT, whilst negotiating the critically intensive learning period. In other words, it will facilitate their competency (actual performance).

This conceptual framework is only the start of a scholarly conversation; it can be used in future research to support data collection and analysis about student preparedness for WCT. This research will be theoretically grounded in a novel way; drawing on many theories to produce a conceptual framework of preparedness for WCT *de novo*. It is only after this validity evidence has been gathered to support its existence should the “Preparedness Toolkit” be considered for implementation in the preparation of students for WCT. A further extension of the conceptual framework would be to generate “tools” for the other members of the community of practice (for example clinical supervisors), and the workplace environment itself, to facilitate students' WCT experience.

## Author Contributions

JR, SP, PC, VN, and KJ contributed to the conception of the manuscript. JR wrote the manuscript. All authors contributed to manuscript revision, read, and approved the submitted version.

## Funding

JR's doctoral studies, to which this work contributes, is sponsored by the Longhurst Legacy (University of Surrey).

## Conflict of Interest

The authors declare that the research was conducted in the absence of any commercial or financial relationships that could be construed as a potential conflict of interest.

## Publisher's Note

All claims expressed in this article are solely those of the authors and do not necessarily represent those of their affiliated organizations, or those of the publisher, the editors and the reviewers. Any product that may be evaluated in this article, or claim that may be made by its manufacturer, is not guaranteed or endorsed by the publisher.
