# Supplementary material for: Using Learning Theories to Develop a Veterinary Student Preparedness Toolkit for Workplace Clinical Training
Source: Front Vet Sci. 2022 Apr 7;9:833034. doi: 10.3389/fvets.2022.833034 (PMC9021599; doi:10.3389/fvets.2022.833034)
Supplement: Supplementary file 1 [file Presentation_1.pdf]

Supplementary Material

1 Supplementary item one: key terms in situated learning theory

Communities of practice

- Communities of practice describes a set of relationships between people and their activities
- It does not necessarily imply co-presence or socially visible boundaries
- Participants in a community of practice have a shared understanding about what they are trying to achieve through work
- Commnities of practice have 'reproduction cycles': the time taken from being newcomer to a core member or 'old timer'
- In the context of veterinary education and WCT, the community of practice could be applied to the individual practice that the student is placed in, or to the veterinary profession itself

Situated learning

- An aspect of all activity in the community of practice
- Decoupled from teaching, it happens whether there's educational intention or not
- The student is 'decentralised' - teaching is not the central motive of the community of practice, the work is

Legitimate participation

- Legitimate participation describes how the learner engages authentically in the daily function of the community of practice
- Authentic activity is a way for students to not only learn how to be a veterinary surgeon, but they also gain access to what it's like to be one. Learning in the community of practice involves belonging, becoming and identity.
- Students need access to ongoing activity, 'old timers', resources and opportunity for situated learning to take place.
- Activities must be non-menial and transparent so that learners acquire knowledge of how and why activities are performed
- If legitimate participation fails, students become 'sequestered'. This promotes the formation of abstract knowledge with little context and disconnection from the practice culture
- Learning is externalised through participation, this reflects assessment methods where students demonstrate competency
- Language is important; learning involves learning to talk in the manner of full participants, giving the student face validity

Peripheral participation

- Newcomers will have an observational outlook post at first, but learning still occurs through being absorbed in the culture of the practice
- As peripheral participants, students may learn activities of the community of practice in the incorrect order, as less complex, vital or risky tasks are learned first. As an example, students may learn to make skin incisions or suturing before performing difficult surgeries

2 Supplementary item two: key terms in cultural historical activity theory

Outcome

- The overarching common goal of the activity system for example, patient care or student education

Objects

- The immediate purposes of actions which lead to the outcome. Examples might include to improve history taking (object) to provide patient care (outcome)

Divison of labour

- Objects are achieved through the division of labour which is related to the social hierarchy of the community
- For example, the anaesthetist prevents pain and consciousness, the surgeon performs the operation whilst the student learns and the nurse assists. These are separate objects of a shared activity system, achieving an outcome

Subjects

- The individual members of the community engaged in the activities

Community

- The community represents the social context of the activity system

Rules

- The rules are the conventions (workplace knowledge), guidelines or rules that regulate the activities

Tools

- Tools are used by the subjects. They include symbolic artifacts such as the knowledge, competencies and specialist terminology of the community of practice along with the physical tools used to perform the activities such as patients, stethoscopes, diagnostic images, patient management systems or charts
